# Exploring the gut-heart axis: A prospective analysis of microbiota in cardiovascular health

**DOI:** 10.6026/9732063002001900

**Published:** 2024-12-31

**Authors:** Deepika Gnanasekaran, Aadhithya Raaj Pandurangan, Pradeepan Sengiah Ramaswamy, Shrunga Kandhi Raghavendra, Karthick Raman, Gowri Shankar Somasundaram

**Affiliations:** 1 Department of Internal Medicine, Hillingdon Hospital, Uxbridge, London, United Kingdom; 2 Department of Internal Medicine, Madras Medical College, Chennai, Tamil Nadu, India; 3 Department of Internal Medicine, Chamarajanagar Institute of Medical Sciences (CIMS), Kasaba Hobli, Chamarajanagar, Karnataka, India; 4 Department of Cardiology, Government Sivagangai Medical College Hospital, Sivagangai, Tamil Nadu, India

**Keywords:** Gut microbiota, cardiovascular disease (CVD), prospective study, lipid metabolism, inflammation, microbial signatures, microbiome interventions

## Abstract

The relationship between gut microbiota composition and the development of cardiovascular disease, with a potential role of microbial metabolites in inflammatory and metabolic pathways is of interest. We analyzed gut microbiota and markers of cardiovascular health in a cohort of 100 participants for three years to search for microbial signatures correlated with increased CVD risk. Our results show several correlations between specific microbial taxa, lipid metabolism and systemic inflammation, whereby a higher Firmicutes/Bacteroides ratio is associated with a greater incidence of CVD. These results suggest that intervention targeting the microbiome has the potential to reduce risk for CVD and point towards a role for gut microbiota in cardiovascular health.

## Background:

The gut microbiota is a complex community of microorganisms that live in the human gastrointestinal tract and are necessary for several physiological processes, such as immune regulation, metabolism and gut barrier integrity [1, 2]. In the last years, high interest was found regarding the relation between cardiovascular disease and the composition of gut micro-biota. Research shows that the risk of developing a cardiovascular disease could be determined through the microbial metabolites such as short-chain fatty acids (SCFA) and trim ethylamine-N-oxide by altering the metabolic and inflammatory pathways [3, 4]. However, traditional factors such as hypertension, dyslipidaemia and obesity, not to forget lifestyle habits are the greatest contributors to global morbidity and mortality; whereas gut microbiota is progressively becoming considered an emerging participant in the pathogenesis of CVD due to potential modulation in host lipid metabolism, enhancing low-grade inflammatory response and glucose metabolism [5, 6]. New evidence has emerged indicating that an imbalance in gut microbiota composition, referred to as dysbiosis, may lead to an increased risk of cardiovascular events. For instance, a high Firmicutes-to-Bacteroides ratio is linked with obesity and metabolic syndrome, which are precursors of CVD [7]. Some microbial taxa, like Akkermansia, Lactobacillus and Bacteroides, have been shown to confer protective effects against CVD through improving gut barrier function and immune response modulation [8]. This study will expand our knowledge on the involvement of gut microbiota in CVD by prospectively following up gut microbiome profiles as well as markers of cardiovascular health in a cohort of adults. Therefore, it is of interests to that microbial signatures would correlate with risk of CVD and hence could give insight into whether interventions that target the microbiome may be an important tool to reduce risk for cardiovascular disease [9, 10].

## Materials & Methods:

## Study design:

This was a three-year prospective study involving 100 adult participants without pre-existing cardiovascular conditions, selected from a larger cohort in three urban medical centres. The study aimed to assess the link between gut microbiota composition and cardiovascular disease incidence by monitoring changes in microbiota profiles and cardiovascular health parameters annually.

## Participants:

Eligible participants were between 30 and 70 years old, without diagnosed CVD at baseline and agreed to participate in annual follow-ups for three years. Exclusion criteria included recent antibiotic use (within the past 6 months), chronic gastrointestinal diseases, immune disorders and lifestyle factors incompatible with study protocols (e.g., unwillingness to adhere to dietary guidelines). Baseline demographic and clinical data, including body mass index (BMI), blood pressure, lipid levels and inflammatory markers, were recorded.

## Gut microbiota analysis:

Participants provided stool samples at baseline and annually. 16S ribosomal RNA sequencing was used to assess microbial diversity and composition. Key microbial indices included the Shannon Index, Simpson Index and Firmicutes-to-Bacteroides ratio. Specific taxa were analyzed to evaluate associations with known CVD risk factors, such as elevated lipid levels and inflammation.

## Cardiovascular assessment:

Cardiovascular health indicators included blood pressure, lipid profiles (LDL, HDL and triglycerides), inflammatory markers (C-reactive protein and IL-6) and fasting glucose levels. Data were collected through physical examinations and blood tests at each follow-up. Incident cardiovascular events, such as myocardial infarction or stroke, were recorded for those who developed CVD during the study period.

## Statistical analysis:

Associations between gut microbiota composition and CVD risk were evaluated using Cox proportional hazards models, adjusting for confounders such as age, gender, BMI and lifestyle factors. Pearson correlation coefficients were calculated for microbial diversity and cardiovascular biomarkers. Statistical significance was set at p<0.05, with analyses performed using R software.

## Results:

The Table 1 outlines the baseline demographic and clinical characteristics, illustrating the relatively healthy profile of the cohort, with average BMI and lipid profiles within the normal range. A small percentage of participants had borderline elevated blood pressure and cholesterol levels. In Table 2 Microbial diversity, represented by the Shannon and Simpson indices, demonstrated slight declines over time, particularly in participants who experienced cardiovascular events, suggesting an association between lower diversity and increased CVD risk. Certain microbial taxa in Table 3 exhibited significant correlations with cardiovascular risk markers. Bacteroides and Akkermansia were inversely associated with lipid levels, while Firmicutes showed a positive correlation with inflammation markers. Table 4 represents, a higher Firmicutes-to-Bacteroides ratio was associated with increased cardiovascular event incidence, suggesting that this ratio may serve as a biomarker for cardiovascular risk. Table 5 shows the adjusted hazard ratios for CVD risk indicated a significant association between a high Firmicutes-to-Bacteroides ratio and cardiovascular events, even after accounting for confounding variables.

## Discussion:

Our findings demonstrate a significant relationship between gut microbiota composition and cardiovascular disease risk, corroborating previous research suggesting that microbial imbalances may contribute to cardiovascular pathogenesis [11, 12]. Specifically, a higher Firmicutes-to-Bacteroides ratio was associated with an elevated risk of CVD, aligning with studies that link this ratio to systemic inflammation, lipid dysregulation and metabolic syndrome [12, 13]. These microbial shifts may exacerbate cardiovascular risk by altering lipid absorption, promoting low-grade inflammation and reducing the production of beneficial metabolites like SCFAs [13, 14]. Lower microbial diversity, as indicated by a reduced Shannon Index, also correlated with higher CVD incidence in our study [15, 16]. This is consistent with previous studies that report lower diversity as a predictor of metabolic disorders, including type-2 diabetes and obesity, both of which are established risk factors for CVD [17, 18]. Altered microbial diversity can impair gut barrier integrity, leading to translocation of pro-inflammatory bacterial components, such as lipopolysaccharides, into systemic circulation, which may promote atherosclerosis [19, 20]. While these findings highlight potential microbial targets for CVD prevention, limitations exist, including the observational nature of this study and potential confounders that may influence both microbiota composition and cardiovascular outcomes [21, 22]. Future research should include randomized trials to establish causality and explore the efficacy of microbiome-modulating interventions, such as prebiotics, probiotics and dietary changes, in reducing CVD risk [23-24]. This article reviews the normal function and composition of the gut microbiome, mechanisms leading to the leaky gut syndrome, its mechanistic link to CVD and potential novel therapeutic approaches aimed towards restoring gut microbiome and CVD prevention [25]. The gut microbiota is emerging as a potential therapeutic target for CVD prevention and management. However, current research has limitations, including the need for larger and more diverse studies, the challenges of establishing causality and concerns regarding the long-term consequences and safety of gut microbiota modulation [26].

## Conclusion:

The potential of gut microbiota composition, particularly a high Firmicutes-to-Bacteroides ratio and reduced diversity, as predictors of cardiovascular disease risk is of interest. These findings suggest that gut microbiota markers could aid in identifying individuals at higher CVD risk and underscore the potential of lifestyle and dietary interventions for prevention. Further research is needed to elucidate the mechanisms and develop personalized microbial therapies for CVD management.

## Figures and Tables

**Table 1:** Baseline characteristics of study participants

**Characteristics**	**Value**
Sample size	100
Mean age	52.3 ± 11.5 years
Gender ratio (Male/Female)	49%/51%
Mean BMI	26.8 ± 4.9 kg/m^2^
Baseline hypertension (%)	15%
Baseline dyslipidemia (%)	20%

**Table 2:** Microbial diversity indices over time

**Microbial Diversity Index**	**Baseline**	**Year 1**	**Year 3**
Shannon Index	4.3	4.1	3.8
Simpson Index	0.85	0.82	0.76

**Table 3:** Association between microbial taxa and cardiovascular risk markers

**Microbial Taxa**	**Lipid Profile Correlation (r)**	**Inflammatory Marker Correlation (r)**
Bacteroides spp.	-0.51	-0.33
Firmicutes spp.	0.48	0.46
Akkermansia spp.	-0.29	-0.24

**Table 4:** Incidence of cardiovascular events by microbiota composition

**Microbiota Composition**	**CVD Incidence (%)**
High Firmicutes/Bacteroides	12%
Low Firmicutes/Bacteroides	5%

**Table 5:** Multivariate analysis: microbiota and CVD risk

**Variable**	**Hazard Ratio (95% CI)**
Firmicutes/Bacteroides ratio	1.42 (1.10-1.85)
Shannon Index	0.84 (0.69-0.97)
